# A Comprehensive Bibliographic Review Concerning the Efficacy of Organic Acids for Chemical Peels Treating Acne Vulgaris

**DOI:** 10.3390/molecules28207219

**Published:** 2023-10-22

**Authors:** Șoimița Emiliana Măgerușan, Gabriel Hancu, Aura Rusu

**Affiliations:** Department of Pharmaceutical and Therapeutic Chemistry, Faculty of Pharmacy, George Emil Palade University of Medicine, Pharmacy, Science and Technology of Tîrgu Mureș, 540142 Târgu Mureș, Romania; emiliana.magerusan@umfst.ro (Ș.E.M.); aura.rusu@umfst.ro (A.R.)

**Keywords:** chemical peels, acne vulgaris, α-hydroxy acids, β-hydroxy acids

## Abstract

Acne vulgaris stands out as the most prevalent skin disorder among teenagers and young adults, causing physical discomfort and considerable economic and psychological burdens on individuals and society. A wide range of topical and systemic therapies are available in acne treatment. Chemical peeling is a skin resurfacing technique designed to rebuild healthy skin using exfoliating substances, a simple and affordable process with various dermatological uses. Chemical peels, classified as superficial, medium, and deep, have been utilized for acne vulgaris and multiple other skin issues. In these chemical peels, a diverse range of chemical substances is employed, each with its unique mode of action. Among these, α-hydroxy and β-hydroxy acids have gathered attention for their efficacy in reducing acne lesions and enhancing overall skin appearance. Acids, such as salicylic acid, glycolic acid, or lactic acid, are commonly used in chemical peels due to their exfoliating and sebum-regulating properties. Despite the widespread use of these acids, there exists a lack of consensus regarding the most effective acid type and concentration for treating acne-prone skin. This review aims to bridge this knowledge gap by evaluating the effectiveness and safety of various organic acids used in chemical peels specifically for acne-prone skin. The findings of this comprehensive bibliographic review indicate that organic acid-based chemical peels represent effective and safe treatment options for individuals with acne-prone skin. Their adaptability sets these treatments apart; the choice of organic acid can be tailored to meet individual patient needs and tolerability levels. This personalized approach ensures that patients receive optimal care while minimizing the risks associated with the treatment. As research in this field progresses, it is anticipated that a more nuanced understanding of the ideal acid type and concentration will emerge, further enhancing the efficacy and safety of chemical peels for acne-prone skin.

## 1. Introduction

Acne vulgaris is the most common skin disease affecting adolescents and young adults. Although it is seen as a mild skin condition, it places a significant economic and psychological cost on individuals and society. Anxiety, depression, and poor self-esteem are common among patients suffering from acne vulgaris, negatively impacting their quality of life [1,2]. *Propionibacterium acnes* colonization, inflammation, enhanced keratinocyte proliferation in the follicular infundibulum, and hypersensitive androgen-sensitive sebaceous glands are some of the components involved in the pathogenesis of acne. It is a disease of the pilosebaceous units, clinically characterized by seborrhea, comedones, papules, pustules, nodules, cysts, and, in some cases, scarring [3,4].

Acne is not due to a single etiological factor but several causes that affect characteristic lesions. Among the most frequently involved causes of acne, heredity, nutrition, hygiene, stress, hormones, and certain cosmetics products or medicines are worth mentioning [4,5]. There are several grading systems for the assessment of acne severity based on the number and type of lesions present on the skin (Global Acne Grading System (GAGS), Leeds Acne Grading System (LAGS), Pillsbury Acne Grading System, Cook’s Acne Grading Scale), the choice of acne grading system depending on the preference of the dermatologist or clinician and the purpose of the assessment [6,7]. Clinical signs for acne vulgaris include dilated pores, comedones, whiteheads, blackheads, pustules and/or papules, nodules, and microcysts, localized especially on the T-zone, the lower section of the face, mandibular or chin area, and, sometimes, the forehead [4,8].

Topical therapies, systemic therapies, chemical peels, dermabrasion, and light therapy are used in conjunction to treat acne. Acne treatment aims to reduce the number of lesions, prevent scarring, and improve the skin’s overall appearance. The treatment approach may vary depending on the severity and type of acne and individual factors such as age, skin type, and medical history. It is important to note that acne treatment may take several weeks or months to show results [9]. Topical therapies include topical retinoids, benzoyl peroxide, antibiotics, or salicylic acid for mild to moderate acne. These reduce inflammation, unclog pores, and kill bacteria [10]. Systemic therapies include the oral administration of antibiotics, hormonal medications, or isotretinoin; these therapies are used for moderate to severe acne and reduce inflammation, kill bacteria, or regulate hormones [11]. Light therapy uses visible light or lasers to kill bacteria and reduce inflammation, which is appropriate for moderate to severe acne. Extraction involves manually removing comedones, pustules, and cysts and should only be performed by a trained professional to avoid scarring and infection. Also, lifestyle changes should be considered, such as maintaining good hygiene, avoiding certain foods that may exacerbate acne, and managing stress levels [12,13].

Chemical peeling is a skin resurfacing technique frequently used for cosmetic and face rejuvenation. A new epidermal layer of the dermal tissues regenerates because the skin suffers controllable damage under the influence of a chemical agent. The amount of acid employed, the kind of vehicle, the amount of buffering, and the length of skin contact all affect how deep the peeling is. Chemical peels are divided into superficial (epidermis-papillary dermis), medium (papillary to upper reticular dermis), and deep peels (mid-reticular dermis), depending on how deeply they penetrate the skin [14].

Medium-depth and superficial chemical peels are used frequently to treat acne vulgaris, as there are various substances and combinations of peels (alpha-hydroxy acids, beta-hydroxy acids, polyhydroxy acids, and bionic acids). Chemical peels can decrease sebum production and have antibacterial, anti-inflammatory, keratolytic, and comedolytic properties. Consequently, chemical peels have been utilized widely to treat acne vulgaris as an additional or maintenance therapy [15,16].

Although chemical peels are commonly used to treat acne, there is a lack of high-quality studies to support their efficacy and safety. Some small studies have shown promising results. Thus, there is a need for larger, randomized, controlled trials to understand better the effectiveness of chemical peels in treating acne. Additionally, there is a need for more research to determine the optimal type of chemical peel and concentration of the active ingredient for treating acne. The available studies have used a variety of chemical peels, including salicylic acid, glycolic acid, and trichloroacetic acid, and the optimal concentration and duration of treatment may vary depending on the individual and the severity of the acne. Furthermore, there is a need for more research to determine the safety of chemical peels in treating acne, particularly in individuals with darker skin tones who may be at a higher risk of hyperpigmentation or scarring [17,18].

This review’s objective is to evaluate and summarize the existing literature to determine the effectiveness of chemical peels and to identify and categorize the different types of organic acids commonly used in chemical peels for acne treatment.

## 2. Materials and Methods

This comprehensive bibliographic review aims to evaluate the effectiveness and safety of various acid types used in chemical peels to treat acne skin. The authors searched multiple databases (Google Scholar, Science Direct, Scopus, Wiley) for relevant studies published between 2010 and 2022. Inclusion criteria encompassed randomized controlled trials, prospective cohort studies, and retrospective studies evaluating the use of organic acid-based chemical peels for acne management. The selected studies were critically appraised, and data were extracted and synthesized using a narrative approach.

## 3. Organic Acids Used in Chemical Peels for Acne Treatment

Chemical peels use a variety of substances to exfoliate the skin and improve its texture and appearance. The type of substances used depends on the desired level of exfoliation and the skin concerns being addressed. It is important to note that the concentration and pH of the chemical peel solution can also affect the level of exfoliation and the risk of adverse effects. Chemical peels should only be performed by trained professionals who can select the appropriate substance and concentration based on the individual’s skin type and concerns [19].

### 3.1. Classification of the Chemical Peel Agents Based on the Depth of Penetration

Chemical peeling agents can be classified based on the depth of penetration into the skin; there are three main types of chemical peels based on the depth of penetration: superficial, medium, and deep. The characteristics of these types of peeling are presented below.

#### 3.1.1. Superficial Peels

Superficial peels penetrate only the outermost layer of the skin (epidermis) and are used to improve skin texture, reduce fine lines and wrinkles, and treat mild acne. These peels involve the application of a chemical solution to the skin’s surface, which helps to exfoliate dead skin cells, unclog pores, and stimulate the growth of new, healthier skin. These peels use weak acids like α-hydroxy acids (AHAs), such as glycolic acid and lactic acid, and β-hydroxy acids (BHAs), such as salicylic acid, to exfoliate the skin. Superficial peels have minimal downtime and are generally safe for all skin types [19,20].

#### 3.1.2. Medium-Depth Peels 

Medium-depth peels penetrate the epidermis and upper layers of the dermis and treat more severe skin concerns such as sun damage, fine lines and wrinkles, and acne scars. Medium chemical peels are a more aggressive form of chemical peel compared to superficial peels. They are sometimes used to manage acne vulgaris, particularly for individuals with more severe or stubborn acne and acne scarring. These peels use trichloroacetic acid (TCA), Jessner’s solution, or a combination of TCA and glycolic acid to exfoliate the skin. Medium-depth peels have a more extended downtime and may require pain relief treatment during the procedure [21,22].

#### 3.1.3. Deep Peels

Deep peels penetrate the deeper layers of the dermis and are used to treat severe skin concerns such as deep wrinkles, scarring, and severe sun damage. These peels use phenol, TCA, or a combination of TCA and phenol to exfoliate the skin. Deep peels have the most extended downtime and are generally only recommended for fair-skinned individuals due to the risk of hypopigmentation or other adverse effects. Deep peels can also determine the appearance of second-degree burns; applying the solution is preferred based on phenols in this condition. The treatment must be used only once and with the utmost caution due to adverse reactions (the appearance of spots that will not disappear); this method is recommended only for people with severe dermatological problems and should only be performed by a dermatologist [22,23].

#### 3.1.4. Peeling Agents

Among the factors that influence the penetration capacity of the tissues by the chemical substances are the pH of the peeling agent, the number of layers applied, and the time given for the action before the substance is neutralized. The penetration depth is determined by the peeling agent’s nature, concentration, and pH. The most common peeling agents used in different chemical peel types are presented in Table 1 [24].

## 4. Classification of the Chemical Peel Agents Based on Chemical Structure

### 4.1. Aliphatic Carboxylic Acids

#### 4.1.1. Azelaic Acid

**Figure 1:**
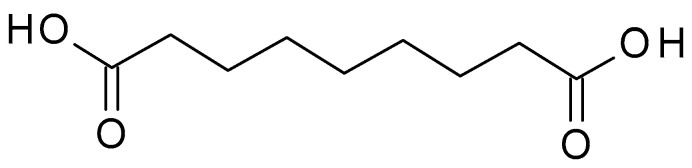
Azelaic acid chemical structure and the IUPAC name: nonanedioic acid.

Azelaic acid (C_9_H_16_O_4_) (Figure 1) is a naturally occurring dicarboxylic acid found in whole grain cereals, wheat, rye, and barley. It is a straight-chain, saturated fatty acid with a carbon chain length of nine carbon atoms. It is a white crystalline powder with a slight odour, sparingly soluble in water but more soluble in organic solvents. The pKa value of azelaic acid is around 4.55, indicating that it exists mainly in its non-ionized form at physiological pH. The pH of azelaic acid formulations typically ranges from 4 to 5, which is close to the skin’s natural pH level [25].

Azelaic acid is used in skincare as a topical treatment for acne and rosacea. The exact mechanism of action of azelaic acid in treating acne is likely multifactorial and involves a combination of antibacterial, anti-inflammatory, and keratolytic effects. It has antibacterial properties, inhibiting the protein synthesis of the *Propionibacterium acnes* species, which helps reduce acne-causing bacteria on the skin. It also has anti-inflammatory properties, which can help to reduce redness and inflammation associated with acne. Azelaic acid works by inhibiting the production of keratin and reducing the production of sebum. On acne skin, azelaic acid appears to reverse the conversion of testosterone to dihydrotestosterone through competitive inhibition of 5α-reductase. Azelaic acid has been shown to help normalize the keratinization process in the skin, which can help prevent the formation of comedones and other types of acne lesions [26,27].

Azelaic acid is associated with a low incidence of cutaneous adverse effects and a lack of systemic toxicity that confer a significant advantage over other agents used to treat acne-prone skin. It is generally considered safe for topical use, with mild side effects such as itching, burning, and dryness reported in some individuals [26]. In addition, azelaic acid combined with other therapies is more efficient in treating acne-prone skin and gives a better clinical outcome [27].

#### 4.1.2. Thricloracetic Acid (TCA)

**Figure 2:**
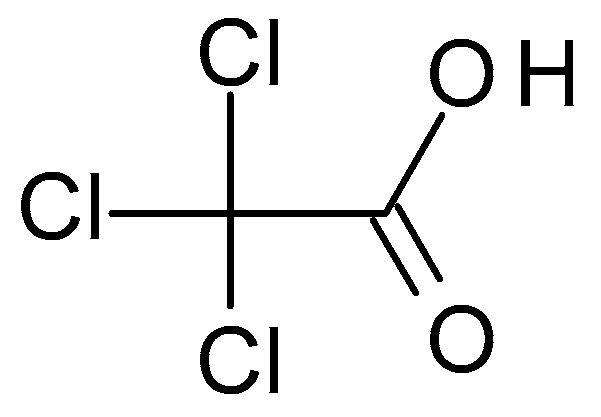
Thricloracetic acid chemical structure and the IUPAC name: 2,2,2-trichloroacetic acid.

TCA (C_2_HCl_3_O_2_) (Figure 2) is a structural analogue of acetic acid in which the hydrogen atoms in the methyl group have been replaced with chlorine atoms. It is a white crystalline solid highly soluble in water and alcohol. TCA has a pH of around 1.5 to 2, which makes it highly acidic [28].

TCA is a highly effective chemical peeling agent for treating various skin conditions, including acne, fine lines, wrinkles, hyperpigmentation, and scars. TCA precipitates epidermal proteins and destroys the upper dermis. Histologically, TCA produces the superficial coagulation of skin proteins and the destruction of the epidermis, followed by the rejuvenation of the epidermis and dermis with new collagen storage and the normalization of the elasticity of the tissue [29]. The depth of the peeling depends on the TCA concentration and the application time. Superficial TCA peeling is performed with concentrations of 10–30%, medium-depth peeling is performed with TCA of 35–50%, and higher TCA concentrations (>50%) should be used with caution due to the risk they present of post-inflammatory hyperpigmentation and scarring. It may be used alone or as a combination peel with other peeling agents [30].

TCA acts by causing a controlled injury to the skin, which stimulates the growth of new skin cells and collagen. This process can help to reduce the appearance of fine lines, wrinkles, and scars, as well as improve the overall texture and tone of the skin [30]. In addition, TCA is a highly caustic and corrosive substance. When misused, it can cause significant skin damage and scarring. TCA cannot be neutralized and has not been associated with allergic reactions or systemic toxicity. During TCA peels, frosting (skin whitening due to protein coagulation) can occur; frosting disappears in 20–30 min and is replaced by erythema, lasting 1–2 days. The redness can last several days up to a week, and peels with TCA can be repeated after 2–6 weeks [30,31].

### 4.2. Aliphatic Hydroxycarboxylic Acids

#### 4.2.1. Glycolic Acid

**Figure 3:**
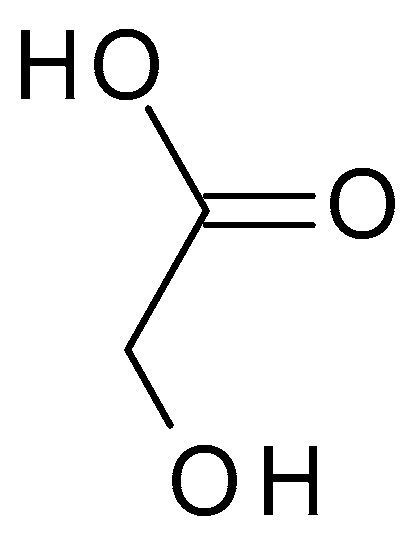
Glycolic acid chemical structure and the IUPAC name: 2-hydroxyacetic acid.

Glycolic acid (C_2_H_4_O_3_) (Figure 3) is the simplest AHA with the smallest molecular weight and size. It is a colourless, odourless, crystalline substance highly soluble in water. This AHA can be isolated from natural sources, such as sugarcane, sugar beets, pineapple, or cantaloupe. Glycolic acid has a pH of around 3.5 to 4, which makes it mildly acidic [32].

Glycolic acid peel is a minimally invasive cosmetic procedure commonly used to treat acne, photo-ageing, and pigmentary disorders such as melasma [33]. Glycolic acid is available in a range of strengths, typically 5% to 30% in skincare products and up to 70% in chemical peels. The peeling will be more intense with the increase in concentration and at lower pH values. At low concentrations (<25%), glycolic acid produces only superficial exfoliation; at concentrations of 25–50%, it causes the discontinuity of keratinocytes, and at concentrations of 50–75%, it produces epidermolysis [34].

Glycolic acid decreases cellular cohesion at the lowest level of the stratum corneum, exfoliating dead cells and encouraging younger, brighter cells to the surface; this superficial exfoliation reduces comedonal acne, pore size, and acne lesions. This process can help improve the skin’s texture and tone, reduce the appearance of fine lines and wrinkles, and improve the absorption of other skincare products [34,35].

This simple AHA can be used in a treatment every 3–4 weeks in a total of 4 to 6 applications. The application time progressively increases depending on tolerance and obtained results after preceding sessions. Glycolic acid can treat acne scars, melasma, post-inflammatory pigmentation, actinic keratoses, fine wrinkles, lentigines, melasma, and seborrheic keratoses [35]. Generally, glycolic acid is considered safe for most patients. However, it can cause skin irritation, redness, and peeling if used at high concentrations or on individuals with sensitive skin [34].

#### 4.2.2. Lactic Acid

**Figure 4:**
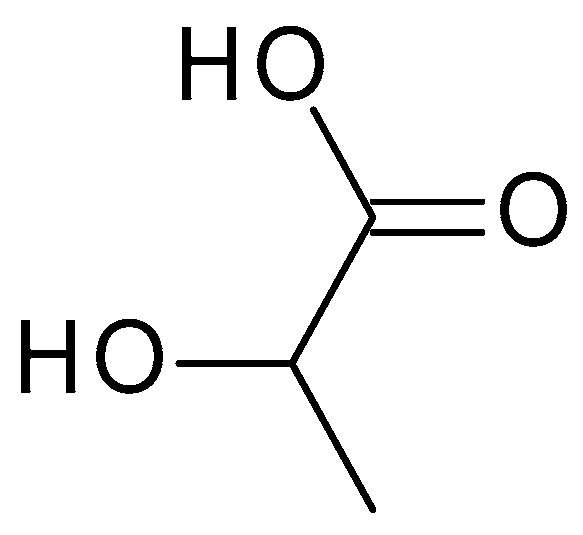
Lactic acid chemical structure and the IUPAC name: 2-hydroxypropanoic acid.

Lactic acid (C_3_H_6_O_3_) (Figure 4) is structurally similar to glycolic acid, having an additional methyl group. Lactic acid is produced via the bacterial fermentation of milk or carbohydrates. It is a colourless or yellowish liquid with a slightly acidic odour. The pH of lactic acid is around 3.5 to 4, which makes it mildly acidic [36].

Lactic acid is available in a range of strengths, typically 5% to 10% in skin care products and up to 50% in chemical peels [37]. Lactic acid acts by loosening the bonds between dead skin cells on the skin’s surface, which helps to exfoliate the skin and stimulate the growth of new skin cells. This process can help to improve the texture and tone of the skin, reduce the appearance of fine lines and wrinkles, and improve the absorption of other skincare products. Lactic acid also has moisturizing properties, which can help to hydrate the skin and improve its barrier function [37].

Chemical peel-based lactic acids are best for persons with sensitive skin who might be unable to tolerate the slightly more intense glycolic acid peel. Lactic acid improves the skin’s photo-ageing, effectively treating post-inflammatory hyperpigmentation lesions in melasma and solar lentigo. Lactic acid inhibits melanin synthesis by inhibiting tyrosinase activity. The treatment starts with low concentrations of lactic acid. The used concentrations should be increased progressively to avoid post-peeling skin hyperpigmentation and irritation [37,38]. Commonly, lactic acid is generally considered safe for most patients; thus, it can cause skin irritation, redness, and peeling at high concentrations or for individuals with sensitive skin [37].

#### 4.2.3. Pyruvic Acid

**Figure 5:**
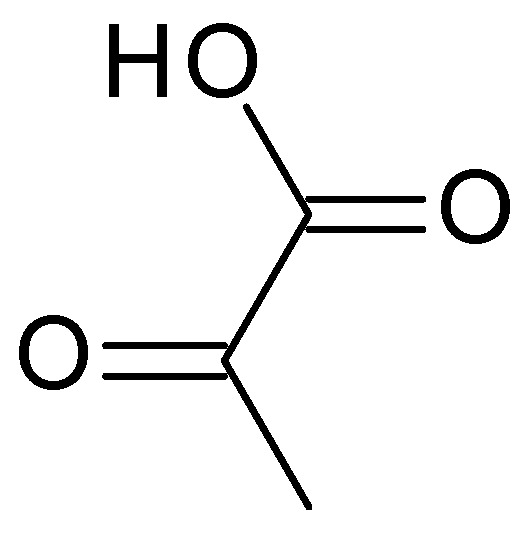
Pyruvic acid chemical structure.

Pyruvic acid (C_3_H_4_O_3_) (Figure 5) is the simplest α-keto acid, with a functional ketone and carboxylic acid group. Pyruvic acid is a colourless to pale yellow liquid with a distinct odour, highly soluble in water and alcohol. Typically, it has a pH range of 2–3 when dissolved in water. It plays a crucial role in various biological processes, including energy metabolism and the synthesis of amino acids [39].

Chemical peels using pyruvic acid primarily focus on exfoliation, reducing sebum production, and improving the overall appearance of acne-prone skin. By promoting exfoliation, pyruvic acid can reduce the build-up of sebum, bacteria, and debris on the skin’s surface, contributing to the development of acne. Due to its anti-inflammatory properties, pyruvic acid can help lessen the redness and irritation associated with acne lesions. In addition to aiding quicker healing and lowering the likelihood of post-inflammatory hyperpigmentation, it can help relax the skin and reduce the inflammatory response. Pyruvic acid has antimicrobial properties that can help inhibit the growth of acne-causing bacteria, such as *Propionibacterium acnes*. Pyruvic acid can lessen the visibility of acne scars, smooth out rough skin, and improve the tone and clarity of the skin by exfoliating the skin and encouraging collagen synthesis [40].

#### 4.2.4. Ascorbic Acid

**Figure 6:**
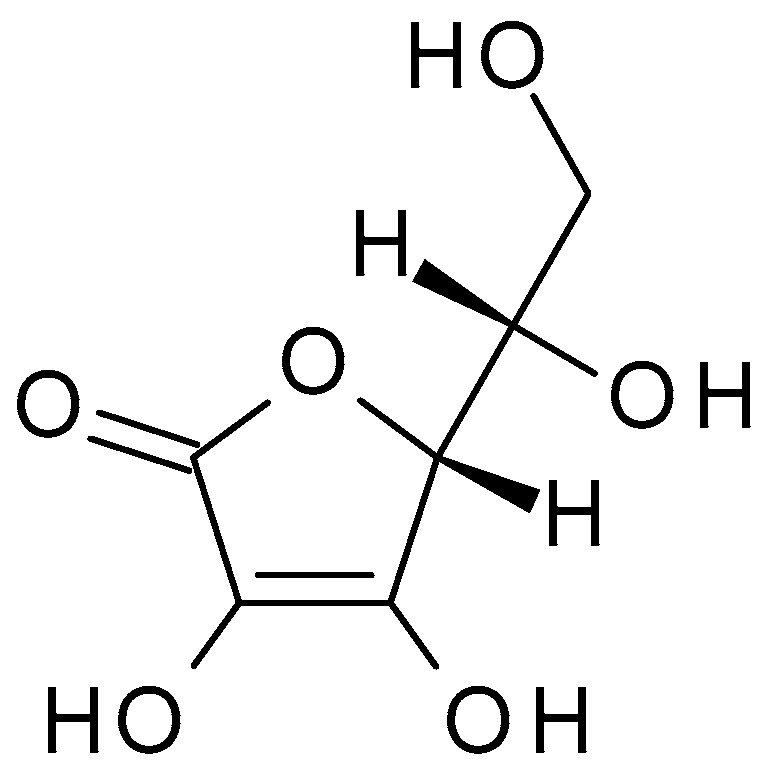
Ascorbic acid chemical structure and the IUPAC name: ((2*R*)-2-[(1*S*)-1,2-dihydroxyethyl]-3,4-dihydroxy-2*H*-furan-5-one).

Ascorbic acid (C_6_H_8_O_6_) (Vitamin C) (Figure 6) is a water-soluble vitamin that plays a crucial role in various bodily functions. Ascorbic acid is susceptible to degradation, particularly under certain conditions such as exposure to heat, light, and oxygen. It is relatively unstable and can undergo oxidation, leading to the formation of dehydroascorbic acid. Ascorbic acid is an organic acid with a relatively low pKa (4.2); it can act as a weak acid, donating hydrogen ions in solution and lowering the pH. It is a good reducing agent and undergoes reversible oxidation-reduction reactions; it can donate electrons, making it an effective antioxidant that can scavenge and neutralize free radicals. Free radicals can contribute to inflammation and damage the skin’s barrier, worsening acne symptoms. It is an essential nutrient; the human body cannot synthesize it, and it must be obtained via diet or supplements. Ascorbic acid helps protect the skin against free radical damage caused by environmental factors such as UV radiation, pollution, and oxidative stress; this antioxidant activity can help prevent premature ageing and support overall skin health [41].

Ascorbic acid has exfoliating properties due to its acidic nature. It acts as a mild chemical exfoliant by breaking down the bonds between dead skin cells, aiding in their removal. By promoting exfoliation, ascorbic acid can unclog pores, remove excess sebum, and reduce bacteria build-up, contributing to the development of acne. This exfoliation process helps improve the skin’s texture, tone, and overall appearance. Chemical peels using ascorbic acid primarily focus on exfoliation, reducing inflammation, and promoting skin healing [42].

Among AHAs derived from fruits, some compounds are useful as secondary ingredients for chemical peels, like citric acid, malic acid, and tartaric acid, which are presented below.

#### 4.2.5. Citric Acid

**Figure 7:**
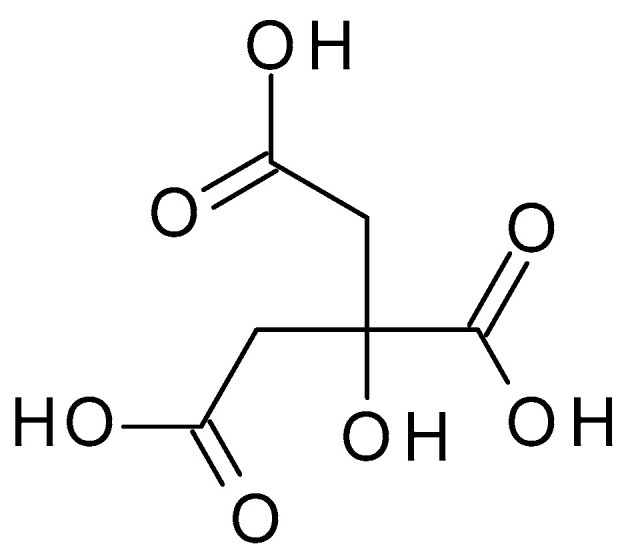
Citric acid chemical structure and the IUPAC name: 2-hydroxypropane-1,2,3-tricarboxylic acid.

Citric acid (C_6_H_8_O_7_) (2-hydroxypropane-1,2,3-tricarboxylic acid) (Figure 7) is a weak organic acid with three carboxylic acid functional groups naturally found in citrus fruits. Citric acid is a white crystalline powder with a tart, acidic taste, which contributes to the sourness of citrus fruits; it is highly soluble in water. Citric acid has a pH value of approximately 2.2 to 2.5 in a 1% aqueous solution. Citric acid has antioxidant properties, donating electrons to neutralize free radicals and prevent their harmful effects [43].

Citric acid helps to exfoliate the top layer of dead skin cells when applied to the skin, accelerating cell renewal and exposing younger-looking skin beneath. Additionally, it can help balance skin tone, cleanse pores, and lessen the visibility of acne scars. However, citric acid can be a powerful peeling agent and might irritate the skin, especially in people with sensitive skin [44].

#### 4.2.6. Malic Acid

**Figure 8:**
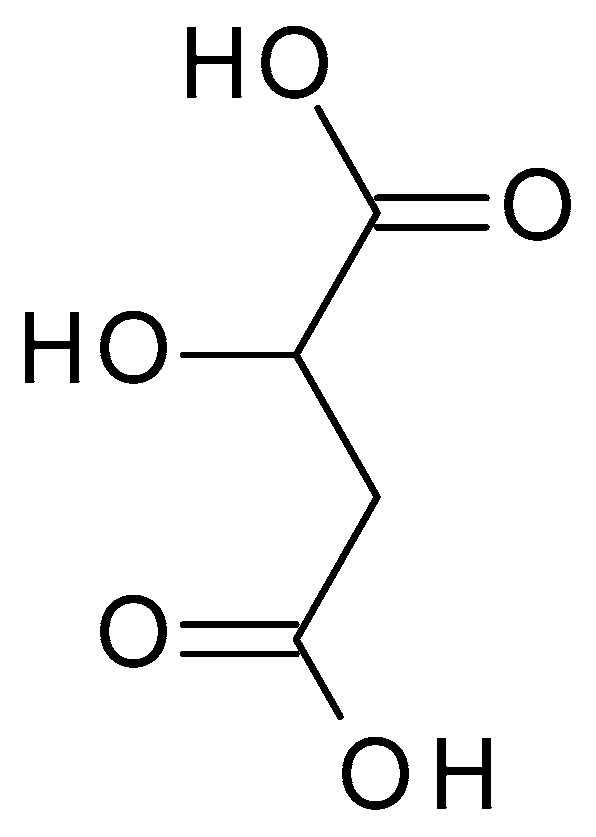
Malic acid chemical structure and the IUPAC name: 2-hydroxybutanedioic acid.

Malic acid (C_4_H_6_O_5_) (Figure 8) is a dicarboxylic acid occurring naturally in various fruits, such as apples, grapes, and citrus. Malic acid is a white crystalline powder with a pleasantly sour taste and is highly soluble in water. Malic acid has a pH value of approximately 2.83 in aqueous solution [45].

Also, malic acid is used in skin care products and chemical peels for its exfoliating and rejuvenating effects on the skin. It helps promote cell turnover, improve skin texture, and enhance overall appearance. Malic acid chemical peels are often softer and more suited for people with sensitive skin or those who want gentler exfoliation. Malic acid tends to have a less potent exfoliating action than AHAs with greater potency, such as glycolic acid [44,46].

#### 4.2.7. Tartaric Acid

**Figure 9:**
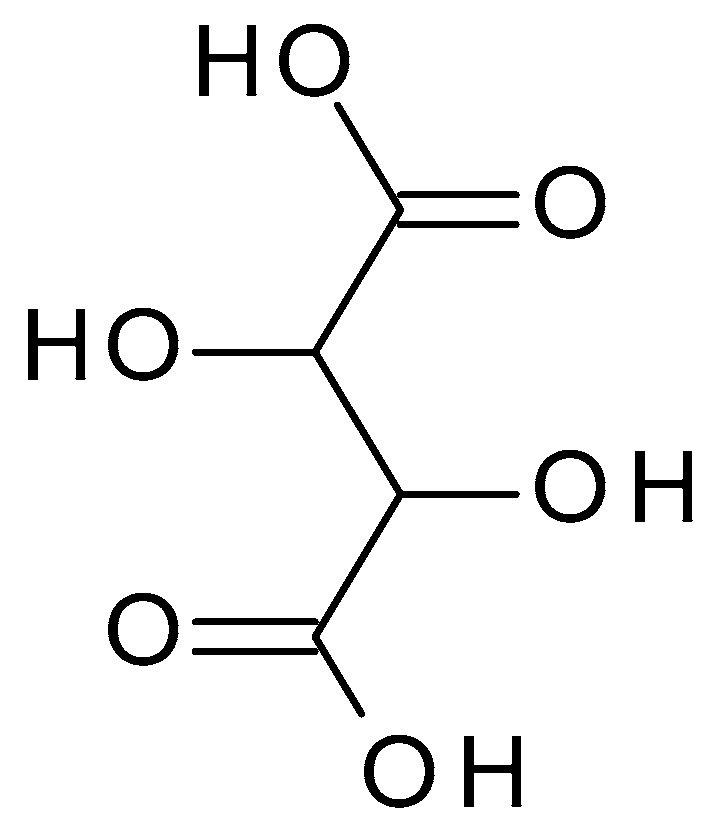
Tartaric acid chemical structure and the IUPAC name: 2,3-dihydroxybutanedioic acid.

Tartaric acid (C_4_H_6_O_6_) (2,3-dihydroxybutanedioic acid) (Figure 9) is a dicarboxylic acid occurring naturally in various fruits, such as grapes or bananas. Tartaric acid is a white crystalline powder with a sour taste; it is highly soluble in water. It has a pH value of approximately 2.2 to 3.0 in aqueous solution [47].

While tartaric acid may have some exfoliating effects, it is not as widely studied or explicitly utilized for treating acne. It can be found as a secondary ingredient or as part of a formulation that combines multiple acids for specific skincare purposes [44,46].

### 4.3. Aromatic Hydroxycarboxylic Acids

#### 4.3.1. Mandelic Acid

**Figure 10:**
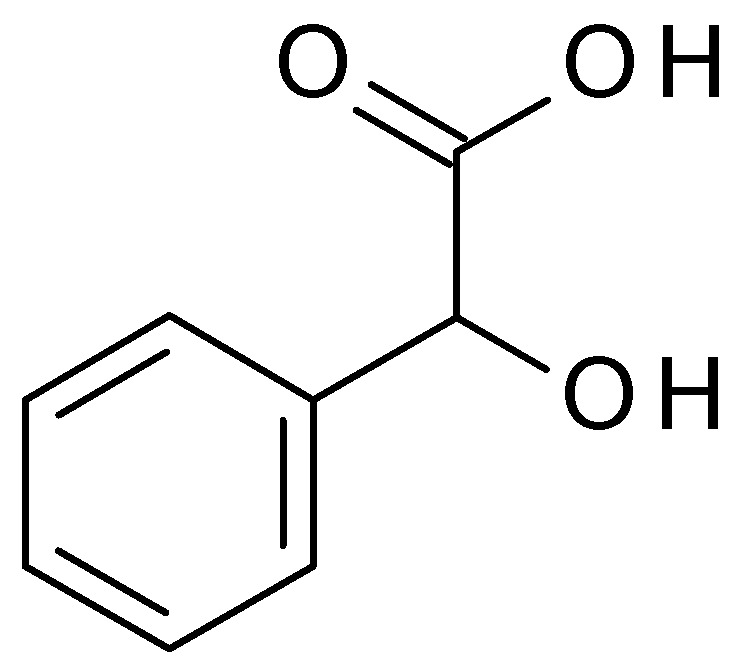
Mandelic acid chemical structure and the IUPAC name: 2-hydroxy-2-phenylacetic acid.

Mandelic acid (C_8_H_8_O_3_) (2-hydroxy-2-phenylacetic acid) (Figure 10) is an AHA derived from bitter almonds. Mandelic acid is a white crystalline powder, highly soluble in water. Mandelic acid has a pH value of approximately 2.8 to 3.2 in aqueous solution [48]. 

Mandelic acid is often used in skincare products for its exfoliating properties; it helps to remove dead skin cells, unclog pores, and improve skin texture. Also, mandelic acid is known for its antibacterial properties, making it beneficial for acne-prone skin. It is less prone to causing skin irritation compared to other AHAs, making it suitable for individuals with sensitive skin [49].

#### 4.3.2. Salicylic Acid

**Figure 11:**
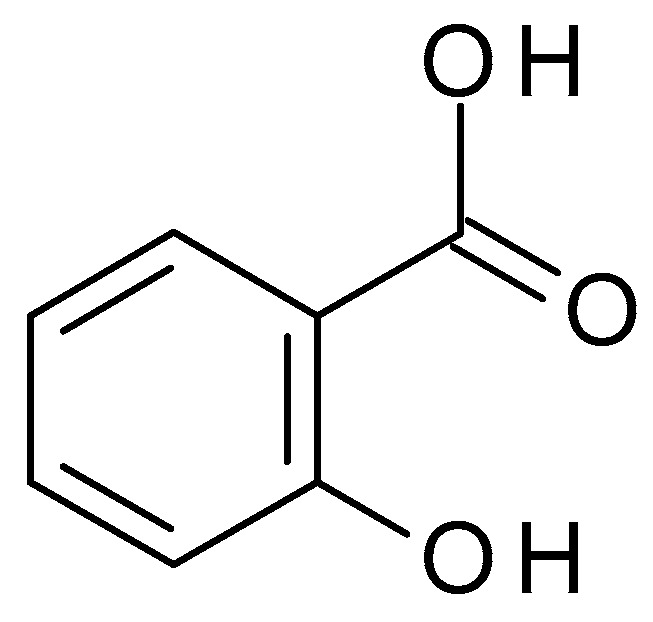
Salicylic acid chemical structure and the IUPAC name: 2-hydroxybenzoic acid.

Salicylic acid (C_7_H_6_O_3_) (Figure 11) is a β-hydroxy acid (BHA) obtained from the bark of the white willow and wintergreen leaves. Salicylic acid is a white crystalline powder, slightly soluble in water but highly soluble in organic solvents. Salicylic acid is a weak acid with a pH value of approximately 2.4 in aqueous solution. It possesses keratolytic, fungicidal, and bacteriostatic effects. Salicylates are acetylsalicylic acid salts and are employed as analgesics [50].

Salicylic acid is commonly used in skincare products for its exfoliating, anti-inflammatory, and acne-fighting properties. As a keratolytic agent, salicylic acid aids in the breakdown and exfoliation of dead skin cells. It gets within the pores and clears out the dirt and sebum that cause acne and blocked pores. Salicylic acid also has anti-inflammatory effects that can help lessen discomfort and redness [51].

Chemical peels with salicylic acid can help improve acne, acne scars, uneven skin tone, and texture. Salicylic acid peels are mainly used to exfoliate the skin, clear clogged pores, and accelerate cell turnover. It works well at eliminating dead skin cells, lowering oil production, and cleaning impurities from the pores [52]. Salicylic acid peels come in various strengths, usually between 20% and 30%. The ideal pH range for salicylic acid to be effective is around 3 to 4; at this pH, it can penetrate the skin and deliver its exfoliating and acne-fighting benefits. The skin type, concerns, and desired amount of exfoliation of the individual determine the peel’s concentration [51,53].

A series of peel treatments may be recommended to achieve the best results, usually spaced several weeks apart. The recovery time required following a salicylic acid peel may vary based on the person’s skin sensitivity and the strength of the peel. While salicylic acid is generally well tolerated, some individuals may experience skin dryness, redness, or irritation, especially if they have sensitive skin. Salicylic acid can increase the skin’s sensitivity to the sun [51,52]. Salicylic acid peels can produce smoother, less acne-prone skin and a more uniform complexion. It can help lessen the visibility of acne scars, lessen hyperpigmentation, and improve the skin’s look [51,53].

#### 4.3.3. Kojic Acid

**Figure 12:**
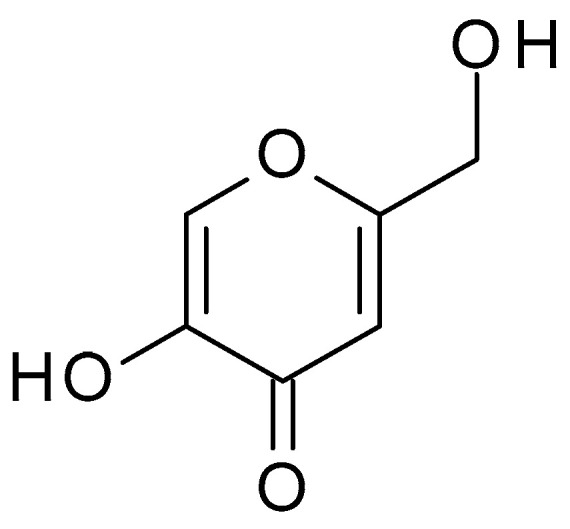
Kojic acid chemical structure and the IUPAC: 5-hydroxy-2-(hydroxymethyl)pyran-4-one.

Kojic acid (C_6_H_6_O_4_) (Figure 12) is a naturally occurring compound derived from several species of fungi, especially *Aspergillus oryzae*. In the fermentation of rice used to make sake (a Japanese alcoholic rice drink), kojic acid is obtained as a byproduct. Kojic acid is a white crystalline powder, sparingly water-soluble, well soluble in organic solvents. It is more stable in acidic conditions (pH 3–5) and less stable in alkaline conditions; it can undergo degradation when exposed to heat, light, and air. Kojic acid has chelating properties, which means it can bind to metal ions such as copper and iron. This property contributes to its ability to inhibit the activity of tyrosinase, an enzyme involved in melanin production. It is commonly used in the cosmetic and skincare industry for its skin-lightening and brightening properties [54,55].

The kojic acid peels can exfoliate the outer layers of the skin, removing dead skin cells, unclogging pores, and preventing the build-up of debris that can contribute to acne formation. Also, these peels can help regulate oil production by exfoliating the skin and reducing the accumulation of sebum in the pores. Kojic acid has some antibacterial properties, which can help combat the growth of acne-causing bacteria, such as *Propionibacterium acnes*, minimizing inflammation and the formation of new acne lesions [56].

Acne breakouts can often lead to post-inflammatory hyperpigmentation, the darkening of the skin in the affected areas. Kojic acid’s skin-lightening properties can help fade these dark spots and promote a more even skin tone [56].

### 4.4. Polyhydroxy Acids (PHAs)

PHAs are a group of chemical compounds used in skincare peels. PHA peels offer a milder alternative to traditional chemical peels, providing exfoliation and various skin benefits [57].

PHA peels provide gentle skin exfoliation, helping remove dead skin cells, unclog pores, and promote cell turnover. PHAs have larger molecular sizes than AHAs and BHAs, making them less likely to penetrate deeply into the skin. As a result, PHA peels are generally considered gentler and more suitable for sensitive skin types [58]. PHAs are frequently combined with additional compounds, including antioxidants, soothing agents, or hydrating components, to maximize the overall effects of the peel and reduce any potential irritation or dryness [57].

Generally, PHAs are well-tolerated by different skin types, including sensitive skin, as they have a lower potential for causing irritation or stinging sensations than other peeling agents. PHAs have humectant properties, meaning they can attract and retain moisture in the skin. Consequently, the PHAs can help improve skin hydration and reduce dryness or flakiness commonly experienced after chemical peels. Often, PHA peels are recommended for individuals with dry or dehydrated skin [58].

The most used PHAs are galactose, gluconolactone, and lactobionic acid, briefly presented below.

#### 4.4.1. Galactose

**Figure 13:**
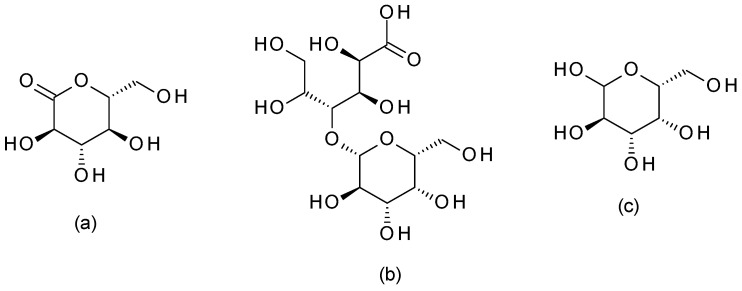
Polyhydroxy acids chemical structures ((**a**)—gluconolactone; (**b**)—lactobionic acid; (**c**)—galactose).

Galactose ((3*R*,4*S*,5*R*,6*R*)-6-(hydroxymethyl)oxane-2,3,4,5-tetrol) (Figure 13) is a PHA sugar molecule. It offers mild exfoliation and can help improve skin hydration and texture. Galactose has humectant properties, helping attract and retain skin moisture [59].

#### 4.4.2. Gluconolactone

Gluconolactone ((3*R*,4*S*,5*S*,6*R*)-3,4,5-trihydroxy-6-(hydroxymethyl)oxan-2-one) (Figure 13) is a PHA derived from glucose. This PHA has a larger molecular size than other hydroxy acids, making it gentle on the skin and offering exfoliation, hydration, and antioxidant properties. It can help improve the skin’s barrier function [59].

#### 4.4.3. Lactobionic Acid

Lactobionic acid ((2*R*,3*R*,4*R*)-2,3,5,6-tetrahydroxy-4-[[(2*S*,3*R*,4*S*,5*R*,6*R*)-3,4,5-trihydroxy-6-(hydroxymethyl)-2-tetrahydropyranyl]oxy]hexanoic acid) (Figure 13) is another PHA commonly used in peels derived from lactose, and it offers similar benefits to gluconolactone. Lactobionic acid provides gentle exfoliation, moisturization, and antioxidant effects and can help improve skin texture and tone [60].

## 5. Combined Peeling Formulas

Combined peeling formulas often utilize a combination of different acids to provide comprehensive exfoliation and address multiple skin concerns. The specific combinations of acids can vary depending on the desired outcomes and the individual’s skin type and needs. Some peeling formulas combine multiple acids in specific ratios to provide a balanced and customized approach. For example, a combination peel may include a blend of AHAs, BHAs, and PHAs to simultaneously address various skin concerns, such as acne, hyperpigmentation, and texture [61]. Usually, a mixture of AHAs and beta-hydroxy acids BHAs, such as glycolic acid, lactic acid, and salicylic acid, can target multiple aspects of acne. AHAs exfoliate the skin’s surface, while BHAs penetrate the pores to unclog them. This combination can help with acne-related concerns like uneven skin tone [62].

Salicylic acid and glycolic acid peel effectively treat active acne and exfoliate the skin. Salicylic acid penetrates the pores to remove excess oil and unclog them. In contrast, glycolic acid exfoliates the outer layer of the skin, promoting cell turnover and reducing the appearance of acne scars. This combination can improve skin texture and reduce acne breakouts [63]. Salicylic acid combined with mandelic acid peel combines the pore-clearing and anti-inflammatory properties of salicylic acid with the gentle exfoliation and antibacterial effects of mandelic acid, helping to unclog pores, reduce acne-causing bacteria, and improve overall skin texture [64].

Salicylic acid and trichloroacetic (TCA) peel can be used for more severe acne and scarring. TCA is a stronger peeling agent that can penetrate deeper layers of the skin, stimulating collagen production and improving the appearance of deeper scars, while salicylic acid complements TCA by targeting active acne and surface imperfections [65]

Also, salicylic acid combined with retinol peeling formula incorporates the pore-clearing benefits of salicylic acid with the skin-renewing effects of retinol. It helps unclog pores, regulate oil production, and promote cellular turnover for smoother skin [66].

Glycolic acid combined with lactic acid and azelaic acid peeling formula incorporates multiple AHAs to exfoliate the skin, reduce acne scars and hyperpigmentation, and promote cell turnover. Azelaic acid further contributes to its antibacterial and anti-inflammatory properties, making it efficient for acne-prone skin [67].

Another example is the combination between mandelic acid and azelaic acid peel. Mandelic acid is an AHA that is less irritating than glycolic or lactic acid, making it suitable for sensitive skin. In contrast, azelaic acid is antibacterial and effective against acne-causing bacteria. This combination can help reduce acne breakouts, improve skin texture, and address pigmentation issues [68].

The most popular combined peeling formula is Jessner’s peel, named after the American dermatologist Dr. Max Jessner, who developed the formula in the early 20th century. This peeling formula can address various skin concerns, including acne, acne scars, sun damage, fine lines, and uneven skin tone. It is a medium-depth peel combining three key ingredients: resorcinol, lactic acid, and salicylic acid. The specific concentrations of these ingredients in a Jessner’s peel can vary depending on the patient’s skin type and the desired results. Usually, the composition of the formula is customized to meet individual needs, adjusting the strength of each component as necessary [69].

Jessner’s peel exfoliates the outer layer of the skin, helping to remove dead skin cells, unclog pores, and clear away debris, preventing the formation of comedones and reducing the occurrence of acne breakouts. Salicylic acid, one of the main components of Jessner’s peel, can penetrate the pores and dissolve excess oil. It can help prevent the accumulation of sebum. The combination of lactic acid and salicylic acid provides anti-inflammatory properties. In addition, this combination can help to calm existing acne lesions, reduce redness, and alleviate inflammation. Jessner’s peel promotes the regeneration of new skin cells and stimulates collagen production, resulting in improved skin texture, smoother appearance, and a more even skin tone. Also, it can help reduce the appearance of acne scars and hyperpigmentation caused by acne. Jessner’s peel is a controlled treatment that can be adjusted based on the individual’s skin type and condition. The applied number of layers and the duration of the peel can be customized to address specific acne concerns and minimize the risk of adverse effects [70,71,72].

## 6. Comparative Studies between the Effectiveness and Safety of Different Acids in Chemical Peels on Acne Skin

Several comparative studies were reported concerning the effectiveness of different acids in chemical peels for acne skin. Also, these studies provided valuable insights into their relative benefits. It is vital to remember that several variables, including the acid’s concentration and pH, the treatment technique, and specific patient characteristics, might affect how effective acids are in chemical peels.

Kessler E. et al. (2008) compared AHA and BHA peels to treat mild to moderately severe facial acne; a split-face, double-blind, randomized, controlled study with 20 participants was conducted. Every two weeks, 30% glycolic acid was applied to one-half of the face, and 30% salicylic acid was applied to the other half for six treatments; the study was conducted for two months. A blindfolded evaluator quantitatively evaluated papules and pustules. By the second treatment session, both peels proved effective, and there was no significant difference between their efficiency. At two months post-treatment, the salicylic acid peel had sustained its effectiveness. After the initial treatment, more adverse events were reported with the glycolic acid peel. Both glycolic acid (AHA) and salicylic acid (BHA) peels were similarly effective in treating mild to moderately severe facial acne vulgaris. However, salicylic acid peel exhibited sustained effectiveness and had fewer reported side effects [73].

Jae J. et al. (2018) compared the efficiency of buffered 50% glycolic acid (pH 3.0) combined with 0.5% salicylic acid and Jessner solution in acne peels. A split-face, evaluator-blind, randomized, controlled study including 20 participants was conducted. The buffered glycolic acid was applied to one-half of the face, and the Jessner solution was applied to the other half. Each patient completed two sessions of treatments separated by two weeks; the number of lesions, the severity of the acne, subjective effectiveness evaluation, and side effects were assessed. The two sides had no significant difference in total lesion count, acne severity, or subjective effectiveness rating. The buffered glycolic acid side had fewer side effects than the Jessner’s solution side. The study suggests that chemical peeling using a buffered glycolic acid solution can be as effective as conventional peeling using Jessner’s solution in treating acne vulgaris. This buffered solution may offer the convenience of not requiring neutralization after application [74].

### 6.1. Comparative Studies that Used Azelaic acid Versus Other Peeling Agents

Abdel Hay et al. (2019) compared the efficacy of a combined solution of 20% azelaic acid and 20% salicylic acid versus a 25% TCA peel for treating acne. The study included 34 participants who received four treatment sessions two weeks apart. One side of each patient’s face was treated with the combined solution of salicylic acid and azelaic acid, while the other side was treated with a 25% TCA peel. The results were assessed based on physician-reported clinical improvement, a dermoscopic assessment of erythema, and patient satisfaction. By the end of the study, both treatments led to a significant improvement in acne, with no significant difference in clinical improvement. Patients reported more discomfort on the TCA-treated side. The combination of salicylic acid and azelaic acid is recommended for early-stage treatment if patients have more inflammatory lesions. In comparison, TCA is recommended if patients have more non-inflammatory lesions. Patients reported greater satisfaction with the salicylic acid–azelaic acid-treated side, suggesting that this combination may offer a more comfortable and satisfactory treatment experience than TCA [75].

Chilicka K. et al. (2020) studied the differences in the effectiveness of azelaic and pyruvic acid peels in treating acne vulgaris. The study included 120 young female participants with mild to moderate papulopustular acne who received six peeling sessions at two-week intervals. The study used a parallel clinical design where one group of participants was treated with azelaic acid, and the other group received pyruvic acid treatments. Following the peeling sessions, the clinical examination of the patients revealed a significant reduction in acne severity symptoms in both the azelaic and pyruvic acid groups. There was also an effect in terms of lowering desquamation and skin oiliness. Pyruvic acid was more effective in reducing greasy skin than azelaic acid [76].

### 6.2. Comparative Studies that Used Glycolic Acid Versus Other Peeling Agents

Kim S.W. et al. (1999) compared the efficiency and safety of glycolic acid and Jessner peels on mild to moderately severe facial acne. Subsequently, 26 individuals were treated simultaneously with 70% glycolic acid and Jessner’s solution twice a week on each side of the face. A blinded evaluator, who did not know the randomization code, conducted evaluations of the therapy on the randomized treatment sides. All participants were questioned about how their facial acne had improved and if they encountered adverse effects; they also responded to the preference test for the two peeling techniques. Most patients’ acne severity did not alter after the first therapy session. However, 50% of the patients who received either glycolic acid or Jessner’s solution peels after the third session exhibited improvement. Both peels showed improvement in acne after multiple sessions, but Jessner’s solution had more side effects like erythema and exfoliation [77].

Garg V.K. et al. (2009) compared the efficiency and tolerability of 35% glycolic acid peels and 20% salicylic acid–10% mandelic acid combination peels in active acne and post-acne scarring and hyperpigmentation. The study included 44 participants with facial acne, post-acne scarring, and hyperpigmentation, which were divided into two groups: glycolic acid peels and salicylic acid–mandelic acid combination peels were administered alternately every two weeks for six sessions to one group and the other. Subjective evaluations were conducted by the participants, the attending physician, and a third-party observer; also, both peels’ side effects were documented. Salicylic acid–mandelic acid peels were more efficient for active acne lesions and hyperpigmentation, with fewer side effects. In conclusion, an AHA and BHA combination peel may be more effective and tolerable for people with acne than the more popular AHA peels alone [78].

Ilknur T. et al. (2010) compared the effects of glycolic and amino fruit acids on acne. For this purpose, 24 participants were involved in a single-blind, randomized, two-face comparison research. Peels were administered every two weeks for six months. Additionally, patient preference between the two peeling procedures and evaluations of cutaneous tolerability during application were conducted. The number of non-inflamed lesions after the first month with glycolic acid peels and after the second month with amino fruit acids decreased significantly. Both peels effectively treated comedonal acne, but amino fruit acid peel was more tolerable and less painful than glycolic acid peel. The study suggests that amino fruit acid peels can be a suitable alternative to glycolic acid peels for treating acne vulgaris, with better tolerability and faster concentration escalation. [79].

El Refaei et al. (2015) evaluated the efficacy and tolerability of a combination of 20% salicylic–10% mandelic acid peel compared to a 35% glycolic acid peel in the treatment of active acne vulgaris, post-acne scarring, and associated hyperpigmentation. The study included 40 participants with facial acne vulgaris who were randomly divided into two groups and underwent seven peeling sessions every two weeks. Both peels effectively treated inflammatory acne, noninflammatory acne, post-acne hyperpigmentation, and certain acne scars. Salicylic acid–mandelic acid peel showed higher efficacy in several acne parameters than glycolic acid peel [80].

Sarkar R. et al. (2016) compared 35% glycolic acid, 20% salicylic–10% mandelic acid, and 50% phytic acid peels in treating active acne and post-acne pigmentation. The study included 90 participants with melasma randomly assigned to three groups, each receiving a different chemical peel. A lesion count was performed at the first evaluation and each subsequent appointment, and post-acne hyperpigmentation index and acne score were recorded. After 12 weeks, there had been a noticeable decrease in inflammatory and noninflammatory lesions and substantial post-acne hyperpigmentation index reduction in all three research groups. The study concludes that both glycolic acid and salicylic–mandelic acid peels are equally effective and safe treatment modalities for melasma. These two peels were more effective than phytic acid peels [49].

Rafique S. et al. (2020) compared the clinical efficiency of 35% glycolic acid and 20% salicylic acid peels in post-acne scarring. One hundred participants were involved in the split-face study, and six treatments were applied at two-week intervals. The study employed a randomized controlled trial design; participants with post-acne scarring were randomly assigned to two groups: one receiving a salicylic peel and the other receiving a glycolic peel. Both peels proved their effectiveness in post-acne scarring treatment. However, salicylic acid showed a slight superiority over glycolic acid [81]. 

Also, in a split face study, Manjhi M. et al. (2020) studied the comparative efficacy of 50% glycolic acid peel and 30% salicylic acid peel. Thirty participants were involved in the study and underwent six sessions every two weeks, with one side of the face treated with glycolic acid and the other with salicylic acid. Both peels were effective, but salicylic acid peel was more effective and responded faster in treating mild to moderate acne vulgaris. This enhanced effectiveness of the salicylic acid peel may be attributed to its lipophilic nature, which allows it to penetrate the sebaceous glands more effectively [82].

In a similar study, Pavithra S. et al. (2022) evaluated the efficiency of 70% glycolic acid and 30% salicylic acid peels. Sixty participants with mild to moderate acne were involved in the study, divided into two groups, one group receiving glycolic acid peel and the other group receiving salicylic acid peel, with four sessions every two weeks. Both peels had comparable effectiveness. Salicylic acid peel demonstrated an advantage of achieving an earlier decrease in lesional count than the glycolic acid peel [83].

### 6.3. Comparative Studies that Used Salicylic Acid Versus Other Peeling Agents

Bae B.G. et al. (2013) compared the efficacy and safety of 30% salicylic acid peels with Jessner’s solution peels in treating mild-to-moderate facial acne. A total of 13 male participants with facial acne were enrolled in the study. One side of each patient’s face was treated with Jessner’s solution, while the other was treated with 30% salicylic acid. Three sessions of peels were administered at 2-week intervals. A blinded evaluator counted noninflammatory and inflammatory lesions before and after each treatment. Inflammatory acne lesion counts did not differ significantly between salicylic acid and Jessner’s solution peels. The study concludes that salicylic acid peels were effective for treating inflammatory acne lesions and were more effective than Jessner’s solution peels for treating noninflammatory acne lesions [84].

Dayal S. et al. (2017) compared the efficacy and safety of 30% salicylic acid with Jessner solution in mild-to-moderate acne vulgaris. A total of 40 participants with mild-to-moderate acne were enrolled in the study and were randomly divided into two groups, one treated with 30% salicylic acid peels and the other with Jessner solution peels, which were conducted two weeks apart for six peels. The study concludes that salicylic peels were more effective than Jessner peels in treating noninflammatory lesions, specifically comedones, and improving mild-to-moderate facial acne vulgaris [85].

Nofal E. et al. (2018) evaluated and compared the clinical efficiency and safety of combination chemical peels versus single peels in treating mild to moderate acne. Forty-five patients were divided into three groups: the first group received progressive peels using modified Jessner’s solution followed by 20% TCA on one side of the face versus 30% TCA on the other side; the second group was treated with a combined peeling formula containing 20% salicylic acid and 10% mandelic acid on one side and 30% salicylic acid on the other; while the third group underwent combination sequential peeling of modified Jessner solution and TCA on one side and 20% salicylic acid and 10% mandelic acid combination peels on the other. All patients had six peeling sessions at two-week intervals and were followed up for three months following the last session. Acne lesions improved considerably on both sides of the face; however, improvement was significantly faster and earlier on the sides treated with combination peels. The study concluded that combination peels achieved a higher and earlier therapeutic response than single peels in treating mild-to-moderate acne [86].

How K.N. et al. (2020) also studied the efficiency and safety of 30% salicylic acid compared to the Jessner solution in patients with acne vulgaris and post-acne hyperpigmentation. A total of 36 participants were involved in a split-face, randomized, double-blinded, controlled trial. Each side of the face was randomly allocated to either the Jessner solution or 30% salicylic acid peels. Subjects underwent treatment once every two weeks for a total of three sessions. The study concluded that Jessner’s solution and salicylic acid were equally effective in treating acne vulgaris and reducing post-acne hyperpigmentation [87].

### 6.4. Comparative Studies that Used Pyruvic Acid Versus Other Peeling Agents 

Zdrada J. et al. (2020) compared the effect of a combined peeling formula containing pyruvic acid with a mixture of glycolic and salicylic acids. Fourteen women with acne were involved in the study. Four treatments were applied at two-week intervals. A preparation containing 50% pyruvic acid was used on one side of the face, while on the other, a mixture of glycolic and salicylic was applied. The skin moisture, sebum secretion, and skin tone were evaluated. It was concluded that pyruvic acid increased skin hydration and reduced skin colour. Regarding acne treatment, therapeutic effects were comparable between the acids and high-concentration pyruvic acid mixture. Using a combination of acids in chemical peels yielded fewer side effects than using a single acid in a high concentration [88].

### 6.5. Comparative Studies that Used Mandelic Acid Versus Other Peeling Agents 

Jartarkar et al. (2017) compared the effectiveness of 20% salicylic acid and 30% mandelic acid peels in treating mild to moderately severe acne vulgaris. Fifty participants with mild to moderately severe acne were randomly divided into two groups. The participants underwent six sessions of peeling treatment at two-week intervals. Salicylic acid peel was more effective in reducing inflammatory and noninflammatory acne lesions than mandelic acid peel. However, mandelic acid peel had fewer side effects and did not lead to postinflammatory hyperpigmentation. These results suggest that salicylic acid may offer slightly better results in improving acne lesions, but mandelic acid could be preferable for individuals concerned about side effects like hyperpigmentation [64].

Dayal S. et al. (2020) studied the efficacy and safety of mandelic acid versus salicylic acid peels in mild-to-moderate acne vulgaris. A total of 50 participants were randomly divided into two groups, with one receiving 30% salicylic peels and the other receiving 45% mandelic peels at an interval of two weeks for six sessions. Both treatments improved acne with approximately identical effectiveness. Both peels improved acne with similar efficacy, but salicylic acid was better for noninflammatory lesions and mandelic acid for inflammatory lesions; the adverse effects reported after mandelic acid peels were less severe [89].

A summary of different comparative studies between the effectiveness and safety of different acids in chemical peels on acne skin is presented in Table 2.

## 7. Complications of Chemical Peels with Acids

While acid chemical peels can effectively treat acne, there are potential complications and side effects, mainly if the peels are not administered correctly or if the skin is not adequately prepared or cared for before and after the treatment. The severity and frequency of side effects can vary depending on factors such as the type and concentration of acids used, individual skin sensitivity, and the skill of the practitioner administering the peel [14,17]. Table 3 shows some common side effects associated with chemical peels for acne.

## 8. Conclusions

Acid chemical peels are a popular treatment option for acne and other skin conditions. It is important to note that the effectiveness of acid peels for acne can vary depending on the severity of the condition and individual skin type.

Chemical peelings involve applying a chemical solution to the skin, which causes the outer layers to peel off, revealing new, smoother skin underneath. Different acids are used in chemical peels, including AHAs, BHAs, or TCAs. Regarding the effectiveness of acid chemical peels for acne, the scientific literature contains limited direct comparative studies evaluating different acids specifically for acne treatment. While there is limited head-to-head comparative data on these acids specifically for acne treatment, several studies have evaluated their effectiveness. The review may have encountered limitations such as variations in study designs, sample sizes, concentrations of organic acids used, and follow-up periods. These variations could influence the overall outcomes and comparison between studies.

AHAs, such as glycolic, lactic, and mandelic, are frequently used in chemical peels. They work by exfoliating the outermost layer of the skin and promoting cell turnover. AHAs can help reduce acne lesions, improve skin texture, and diminish post-inflammatory hyperpigmentation caused by acne. Salicylic acid is the most used BHA in chemical peels. It can penetrate the oil glands and exfoliate the pores, effectively treating acne and blackheads. Salicylic acid also possesses anti-inflammatory properties that can help reduce redness and inflammation associated with acne. TCA is preferred in medium to deep chemical peels and is more commonly employed for acne scars rather than active acne lesions. TCA peels can improve the appearance of acne scars by stimulating collagen production and promoting skin regeneration.

Organic acid chemical peels are effective in treating acne skin. Different organic acids, such as glycolic acid, salicylic acid, mandelic acid, and pyruvic acid, have demonstrated positive results in reducing acne lesions, comedones, and post-acne hyperpigmentation. Some organic acids may excel in treating certain types of acne lesions; for instance, salicylic acid has shown effectiveness in treating noninflammatory lesions (comedones), while mandelic acid has been more effective in treating inflammatory lesions.

In general, acid chemical peels have shown a good safety profile when used in appropriate concentrations and under professional supervision. However, some organic acids, like glycolic acid and Jessner’s solution, may cause more side effects, like erythema and exfoliation, than other acids. Some studies have shown that combination peels involving a mix of different organic acids can be more effective and tolerable than single-acid peels. For example, combination peels with salicylic–mandelic acid or modified Jessner’s solution with TCA have demonstrated faster and more significant improvement in acne lesions. The advancements in formulation, individualized treatment plans and safety protocols make chemical peels viable for addressing a wide range of skin concerns while maintaining patient satisfaction and safety.

As an outcome, acid chemical peels can be helpful in acne treatment, but further research is needed to directly compare the effectiveness of different acids for this specific purpose. There is a need for rigorously conducted and meticulously documented randomized controlled trials to generate robust evidence that can effectively guide clinical practice. New research and innovation in chemical peels are necessary to improve their efficacy, safety, and customization options. Continued investment in high-quality randomized controlled trials is essential in acne treatment and beyond. These trials are pivotal in advancing medical knowledge, enhancing patient care, and ensuring healthcare practices are rooted in the best available evidence.

When determining the most effective formulation and regimen for chemical peeling agents, it is imperative to compare placebo and alternative treatments among different ethnic communities and skin types. Comparative studies, among other active treatments, enable researchers to identify the most effective formulation and regimen, guiding clinicians in making informed decisions about the best course of action for their patients. There is a requirement for standardized outcome measurements in the management of acne vulgaris in order to ensure that the data collected are reliable, comparable, and applicable across various contexts, ultimately leading to improved treatments and better outcomes for patient outcomes.

Future studies should explore the long-term effects of chemical peels, particularly in maintenance treatments and their impact on skin health over time. Other research directions may include targeted and controlled-release formulations, combination therapies with other cosmetic procedures, the development of safer and gentler innovative agents, and increased focus on natural ingredients.

## Figures and Tables

**Table 1:** Peeling agents used in different chemical peel types [16,17,24].

Peel Type	Peel Agent	Depth of Penetration
Superficial	AHA—glycolic acid (30–50%), lactic acid (10–30%), mandelic acid (50%) BHA—salicylic acid (30%) Pyruvic acid (50%) Resorcinol (25–50%) Jessner solution (3–7 coats) TCA (10–35%—1 coat)	Superficial/stratum corneum exfoliation/epidermal necrosis Penetrates the outer layer of the skin to exfoliate gently
Medium	AHA—glycolic acid (>70%)—with or without pretreatment Jessner solution BHA—salycilic acid (>30%) TCA (30–50%)—with or without pretreatment Jessner solution TCA 35% + glycolic acid 70%	Medium penetrates the outer layer of the skin Reaches the middle layer of the skin to remove damaged skin cells
Deep	TCA (>50%)—pretreatment Jessner solution Phenol 88% Baker-Gordon phenol peel (50–55% phenol)	Deep/reticular dermal necrosis Reaches deep into the middle layer of skin to remove damaged skin cells

**Table 2:** Summary of comparative studies between the effectiveness and safety of different acids in chemical peels on acne skin (studies are presented chronologically).

Acids	Description	Outcome	Year	Ref.
70% Glycolic acid vs. Jessner solution	26 patients with facial acne; procedures were repeated three times every 2 weeks.	- No significant differences in treatment effects between the two peels; - Jessner’s solution showed a significantly increased degree of exfoliation compared to glycolic acid.	1999	[77]
30% Glycolic acid vs. 30% Salicylic acid	20 patients with mild to moderately severe facial acne; procedures were repeated six times every 2 weeks.	- No significant differences in effectiveness between the two peels; - Salicylic acid peel has sustained increased effectiveness and fewer side effects.	2008	[73]
35% Glycolic acid vs. 20% Salicylic acid + 10% Mandelic acid	44 patients with facial acne and post-acne scarring and hyperpigmentation; procedures were repeated six times every 2 weeks.	- Both agents were effective, with the salicylic acid + mandelic acid combination having better results for active acne and post-acne hyperpigmentation.	2009	[78]
Glycolic acid vs. Amino fruit acid (20%, 35%, 50%, 70%)	24 patients with acne; procedures were repeated for six months every 2 weeks.	- Both agents are efficient for comedonal acne; - Amino fruit acid peel is less irritating and better tolerated than glycolic acid peel.	2010	[79]
30% Salicylic acid vs. Jessner solution	13 patients with facial acne; procedures were repeated six times every 2 weeks.	- Salicylic acid peels were more effective for inflammatory acne than Jessner’s solution peels for treating noninflammatory acne.	2013	[84]
20% Salicylic acid + 10% Mandelic acid vs. 35% Glycolic acid	40 patients with acne vulgaris, post-acne scarring, and associated hyperpigmentation; procedures were repeated seven times every 2 weeks	- Both peels are efficient in treating inflammatory acne, noninflammatory acne, post-acne hyperpigmentation, and certain types of acne scars; - Salicylic acid combined with mandelic acid showed a higher efficacy in several acne parameters.	2015	[80]
35% Glycolic acid, 20% Salicylic acid—10% Mandelic acid vs. 50% Phytic acid	45 patients with active acne; procedures were repeated six times every 2 weeks.	- All agents were efficient in improving active acne, but the inflammatory lesions were better treated with salicylic-mandelic peel.	2016	[49]
30% Salicylic acid vs. Jessner solution	40 patients with mild-to-moderate facial acne; procedures were repeated six times every 2 weeks.	- Salicylic acid was more effective than Jessner solution peels in the treatment of noninflammatory lesions;	2017	[85]
20% Salicylic acid vs. 30% Mandelic acid	50 patients with mild to moderate facial acne; procedures were repeated six times every 2 weeks.	- Salicylic acid peel was more effective in reducing both inflammatory and noninflammatory acne lesions; - Mandelic acid had fewer side effects and did not lead to postinflammatory hyperpigmentation.	2017	[64]
50% Buffered glycolic acid (pH 3.0) + 0,5% Salicylic acid vs. Jessner solution	20 patients with facial acne; procedures were repeated two times every 2 weeks.	- Peeling using the 50% glycolic acid (pH 3.0) + 0.5% salicylic acid solution can be as effective as peeling using Jessner’s solution and shows fewer adverse.	2018	[74]
Modified Jessner’s solution + 20% TCA vs. 30% TCA; 20% Salicylic acid + 10% Mandelic acid vs. 30% Salicylic acid modified Jessner’s solution + 20% TCA vs. 20% Salicylic acid + 10% Mandelic acid	45 patients with facial acne; procedures were repeated six times every 2 weeks.	- Combination peels achieved better responses than single peels.	2018	[86]
20% Azelaic acid + 20% salicylic acid vs. 25% TCA	34 patients with acne; procedures were repeated four times every 2 weeks.	- Both acid peels were efficient in treating acne vulgaris; - More discomfort with TCA.	2019	[75]
35% Glycolic acid vs. 20% Salicylic acid	100 patients with post-acne scarring	- Both acid peels were efficient in treating acne vulgaris - Slightly better results salicylic acid	2020	[81]
50% Pyruvic acid vs. a mixture of glycolic and salicylic acid	14 women with acne; procedures were repeated four times every 2 weeks.	- Pyruvic acid was beneficial for increasing the skin’s hydration and decreasing the skin’s colour.	2020	[88]
Azelaic acid vs. Pyruvic acid	120 young women with facial acne; procedures were repeated six times every 2 weeks.	- Significant reduction of acne severity symptoms for both azelaic acid and pyruvic acid; - Pyruvic acid showed a more significant reduction of greasy skin than azelaic acid.	2020	[76]
30% Salicylic acid vs. 45% Mandelic acid	50 patients with mild to moderate facial acne; procedures were repeated six times every 2 weeks.	- Mandelic acid peel was found to be equally effective as salicylic acid peel;—Safety and tolerability of mandelic acid peel were better than salicylic acid peel.	2020	[89]
30% Salicylic acid vs. Jessner solution	36 patients with mild to moderate facial acne; procedures were repeated three times every 2 weeks.	- Salicylic acid and Jessner solution were equally effective in treating acne and reducing post-acne hyperpigmentation in patients with coloured skin.	2020	[87]
50% Glycolic acid vs. 30% Salicylic acid	30 patients, split-face study, every two weeks peeling sessions	- Both acid peels were efficient in treating acne vulgaris; - Salicylic acid was better tolerated.	2020	[82]
70% Glycolic acid vs. 30% Salicylic acid	60 patients; procedures were repeated four times every 2 weeks.	- Both acids have comparable efficiencies; - Salicylic acid exhibited an earlier decrease in the lesional count.	2022	[83]

**Table 3:** Adverse effects observed after chemical peels with acids on acne skin [14,17,90,91].

Adverse Effects	Management	Comments
Skin irritation and redness	- These symptoms typically subside within a few days to a week; - Application of soothing, hydrating moisturizer to the treated area to alleviate redness and reduce irritation; - Avoid using harsh skincare products or exfoliants until the skin has fully healed.	- Following a chemical peel, it is expected to experience temporary skin irritation, redness, and increased sensitivity; - Individuals with sensitive skin or those who undergo deeper peels may experience more pronounced redness and prolonged irritation.
Post-inflammatory hyperpigmentation	- Proper sun protection and skin-lightening agents may help minimize the risk of post-inflammatory hyperpigmentation.	- Chemical peels can sometimes trigger post-inflammatory hyperpigmentation, especially in individuals with darker skin tones.
Peeling and flaking	- Avoid picking or forcefully removing the peeling skin to prevent potential complications; - Application of a gentle, non-abrasive moisturizer to keep the skin hydrated and minimize dryness; - Avoid harsh cleansers or exfoliants that may further irritate the peeling skin.	- After chemical peels, the treated skin may peel or flak as the outermost layers slough off, resulting in temporary dryness and skin shedding.
Increased sensitivity to sunlight	- It is crucial to protect the treated area from excessive sun exposure and use a broad-spectrum sunscreen with a high SPF to minimize the risk of sunburn and hyperpigmentation; - Sun protection should be continued for several weeks following the peel; - Wearing protective clothing to shield the treated area from the sun further;	- Chemical peels can make the skin more sensitive to sunlight.
Allergic reactions	- It is essential to undergo a patch test before the complete treatment to identify potential allergies.	- Individuals may be allergic or sensitive to certain acids used in chemical peels—can manifest as redness, itching, swelling, or even more severe symptoms.
Infection	- Follow the instructions provided by the dermatologist or skincare professional and keep the treated area clean and protected	- Proper hygiene is not maintained during the treatment, or if the skin is not adequately protected post-peel, there is a risk of infection
Post-treatment complications	- Follow any specific instructions or treatment recommendations provided by the dermatologist to address these complications	- In rare cases, chemical peels can lead to scarring, changes in skin colour, or infection—complications are more commonly associated with deep peels or when the peels are administered incorrectly or by untrained individuals.

## Data Availability

Not applicable.
